# Debated agronomy: public discourse and the future of biotechnology policy in Ghana

**DOI:** 10.1080/11287462.2016.1261604

**Published:** 2017-02-22

**Authors:** Joseph A. Braimah, Kilian N. Atuoye, Siera Vercillo, Carrie Warring, Isaac Luginaah

**Affiliations:** ^a^ Environmental Health and Hazards Lab, Department of Geography, University of Western Ontario, London, Ontario, Canada; ^b^ Department of Geography, University of Western Ontario, London, Ontario, Canada

**Keywords:** Biotechnology, genetically modified organisms, public policy, content analysis, agriculture, Ghana

## Abstract

This paper examines the highly contested and ongoing biotechnology (Bt) policy-making process in Ghana. We analyse media content on how Bt is viewed in the context of Ghana’s parliamentary debate on the Plant Breeders Bill and within the broader public policy-making literature. This paper does not seek to take a position on Bt or the Bill, but to understand how policy actors influence the debate with political and scientific rhetoric in Ghana. The study reveals that in the midst of scientific uncertainties of Bt’s potential for sustainable agriculture production and food security, policy decisions that encourage its future adoption are heavily influenced by health, scientific, economic, environmental and political factors dictated by different ideologies, values and norms. While locally pioneered plant breeding is visible and common in the Ghanaian food chain, plant breeding/GMOs/Bt from international corporations is strongly resisted by anti-GMO coalitions. Understanding the complex and messy nature of Bt policy-making is critical for future development of agricultural technology in Ghana and elsewhere.

## Introduction

Sub-Saharan Africa (SSA) is disproportionally affected by climate change with 27.7% of the 795 million undernourished people globally residing on the continent (FAO, IFAD, & WFP, 2015; Garza & Stover, 2003). To improve agricultural productivity, and thus food security, various agriculture technologies have been proposed, including biotechnology (Bt), innovations in machinery, chemicals, agronomy and information uptake (Godfray et al., 2010; Rosegrant & Cline, 2003). However, deployment of Bt has been highly contested and remains one of the most publically debated technical solutions to food security in some of these countries including Ghana.

Generally, Bt involves an alteration of the genetic make-up of plants through the extraction of desired DNA traits from one organism and introducing it onto another (McAfee, 2003; Stilwell & Van Dyke, 1999). Whereas plants have been going through natural modification or mutation, the present technology of moving individual genes through Bt is more appropriately called genetic engineering (Shelton, Zhao, & Roush, 2002; Stilwell & Van Dyke, 1999). Shelton et al. (2002) identify this agriculture revolution to constitute two forms: genomics, which seeks to understand the organisation of traits within the chromosomes of a species and transgenic, which involves the transfer of individual genes from one species to another. However, much of the public controversy in Ghana is over transgenic technology.

There have been various scientific claims made about Bt, most of which have focused on the safety of changes in food composition caused by genetic modifications, the safety of the inserted DNA and the potential effect of gene(s) transfer (Garza & Stover, 2003; Stilwell & Van Dyke, 1999). While the World Health Organisation maintains that Bt is safe and reduces human health and environmental risks associated with the use of chemical inputs (Kumar, Chandra, & Pandey, 2008; Shelton et al., 2002), there are contrary assertions that it has negative environmental, economic and health impacts (Falkner, 2007). For instance, while Bt cotton has been suggested to promote biodiversity (Carpenter et al., 2002), it has also been cited to create new virus, induce leaching, disrupts the ecosystem and degrades human health among others (Hails, 2000; Peterson et al., 2000). Indeed, several comparative studies conclude that transgenic techniques are not riskier than genomic improvement techniques. These scientific uncertainties and contradictory evidences have generated public concern about the safety of genetically modified organisms for the environment and as food produced for consumption (Carr & Levidow, 2000) especially within the Ghanaian context where traditional plant breeding is the dominant form of agriculture.

Public debate has also centred on the dimensions of business ethics and power imbalances involved in Bt contracts between large multinational corporations and those who could ultimately rely on it (mainly poor and smallholder farmers). This emanated from the fear of losing control over their livelihoods, indigenous seeds and worsening biodiversity (Vercillo, Kuuire, Armah, & Luginaah, 2015). This paper analyses Bt policy claims and debates within Ghana’s Plant Breeders Bill to demonstrate how the acceptance or rejection of agricultural technology is influenced by health, scientific, economic, environmental and political interests. This case study is particularly informative because it contextualises and explains the various scientific claims, ideological and value-laden rhetoric used by differing stakeholders to influence Bt policy development. This paper does not seek to take a position on Bt but to understand how politics and science conjoin to shape the Bt debate in Ghana. This is critical for understanding the future of Bt in Ghana, as it draws attention to the wider policy debate and power dynamics in Africa that influence food security, and global agricultural investment.

## Agriculture policies and technologies in Ghana

Burdened with lower crop yields, smallholder farmers in Ghana are encouraged through government policy strategies to intensify production by adopting improved technology to increase crop yields (Ignatova, 2015). These policies and programmes include the Ghana Poverty Reduction Strategy II, the Food and Agriculture Sector Development Policy, the Savannah Accelerated Development Authority and the Ghana Commercial Agriculture Program. In addition, private programmes such as the Agro-Dealer Development Program have recently been launched as part of the Alliance for a Green Revolution in Africa, supported by the Rockefeller and Bill and Melinda Gates Foundations (Flora, 2010). As part of this initiative, about 2400 agro-dealers are contracted to supply high-yielding seeds and fertilisers to farmers (Nyantakyi-Frimpong & Bezner-Kerr, 2015).

However, some studies have found these input intensive technologies to be poorly suited for the political ecology of agriculture in contemporary Ghana. Awanyo (2001) for instance found in a southern Ghanaian community that smallholders who adopted improved seeds reported increased labour demands on their farms. The new form of hybrid maize production was shown to take up to 55% of the poor farmers’ farm labour, which is deemed to be too high (Awanyo, 2001). In a more recent study, Nyantakyi-Frimpong and Bezner-Kerr (2015) reported that in some communities in northern Ghana, high input use in hybrid maize production has become too costly and labour demanding thereby negatively impacting food security among peasant farmers.

In the midst of complaints regarding hybrid seed technology in Ghana by peasant farmers, the enactment of the Biosafety Act 831 in 2011 and recent attempts to pass the Plants Breeders’ Bill, which would allow Bt application has generated polarised public debate (Adenle, 2014). The Plant Breeders Act seeks to establish a legal framework to promote the breeding of new plant varieties and improve food production in Ghana (Adenle, 2014). Despite adoption of Bt in neighbouring Burkina Faso (Vitale, Glick, Greenplate, & Traore, 2008), the ongoing Bt policy process in Ghana is enveloped by antagonistic claims stemming from the uncertainty regarding the potential of Bt to increase yields without adverse environment and health effects. Ignatova (2015) reports that proponents and opponents of Bt use simplistic and exaggerative claims to garner attention and support, as well as persuasion through “expertise” or science claims. This study unravels the values, ideologies and social structures that inform these simplistic and exaggerative science-based claims by the various stakeholders.

## Theoretical framework

Public policy-making involves a complex interplay of several stakeholders with varied interests or motives. These motives could be as broad as economic policy reform to more specific aims such as election interests and media exposure (Pal, 1992). Hence, this study uses the structural deterministic framework by Pal (1992) and the science-policy claims making discourse to analyse public debate on the Plant Breeders Bill in Ghana. The structural deterministic framework considers social systems and structures as powerful forces influencing individual behaviours and policy decisions (Cook, Emel, & Kasperson, 1991; Pal, 1992). It argues that in human societies, people’s behaviours are governed by underlying systems or structures including their cultural values, norms, ideologies and interrelationships. Thus social, cultural and ideological values serve as significant variables that determine public reaction to the adoption of Bt in Ghana. For instance, Frewer (2003) argue that complexities relating to price, packaging, availability, convenience of Bt and perceived health risks would influence individual acceptance of the technology. Risk perceptions are socially constructed and best determines attitudes towards hazardous exposure rather than the expert led technical estimates (Baxter & Greenlaw, 2005; Frewer, 2003). Also, public opinion about the functions of new technology is dynamic and influenced by information availability on the benefits and risks associated with it (Frewer, 2003; Wynne, 2001). The structural deterministic framework by Pal (1992) is helpful to understand varying discourses and how they shape the debate on Bt acceptance in the specific context of Ghana.

Furthermore, the Plant Breeders Bill debate is a battle between scientific, political and ideological interests. Characterised by complexities and uncertainties, science becomes difficult to distinguish from politically based claims. Garvin and Eyles (1997) attribute this blurred relationship between science and policy to the inability of science to make accurate predictions and find solutions to societal problems. As such, scientists resort to the tools of persuasion and storytelling to influence and inform policy. This reinforces Torgerson’s (1986) third face of policy-making; an emerging trend in policy analysis which espouses a complex blend between science (knowledge) and policy (politics). Thus, this study analyses the application of scientific evidence and politics by different stakeholders using rhetoric to influence Bt policy-making in Ghana.

Moreover, policy-making is ingrained with subsystems comprising of actors from distinct organisations and coalitions who, based on their values and belief systems, push a particular policy agenda using scientific rhetoric (Aronson, 1984; Sabatier, 1987). Claims making becomes the hallmark of policy-making and is used by both scientists and “lay persons” in policy debate. A “lay person” will often use political claims to support their interests, often laced with scientific rhetoric to increase persuasiveness, whereas scientists make cognitive claims which relies on the scientific community to accredit them (Aronson, 1984). Indeed, the relationship between science and policy (politics) as described by Garvin and Eyles (1997) is symbiotic. This paper situates the Plant Breeders Bill public debate in Ghana within the science-policy theoretical discourse to examine and explain the intrinsic complexities and nuances. It intends to provide deeper meaning and understanding of the GMO policy claims and counterclaims in Ghana by seeking answers to the following critical questions: (1) Who are the proponents and opponents of Bt in the debate? (2) What claims do they make and how do they justify these claims? (3) What science, politics and ideologies influence these stakeholders’ choice of forming and joining particular coalitions?

## Methodology

This study uses Howland, Becker, and Prelli (2006) Content Analysis Categorical System Technique which was developed to bridge the methodological gap between content analysis and policy science analytical frameworks by Lasswell (1971). Content analysis is a research approach that subjectively interprets the content of text data through the systematic classification process of coding and identification of themes or patterns (Hsieh & Shannon, 2005). This study uses content analysis to examine the debates on GMO policy decision-making in Ghana. This research approach allows for easy and systematic search through large volumes of data (Howland et al., 2006) on the GMO debate to identify claims and counterclaims by interest groups in the policy debate in a cost effective manner.

### Sources of data

Ghana’s online media platforms served as the main source of data for this study. Ghana’s stable democracy has contributed to a vibrant and competitive media (Bokor, 2015) which serves as a medium for effective and relevant policy debates (Best & Meng, 2015). In this study, newspaper articles on GMOs were gathered from online sources because of their availability and easy access. The Ghanaian online media comprises both private and public portals. A combined extensive search was therefore conducted on both private (myjoyonline.com, modernghana.com, ghanaweb.com and citifmonline.com) and public (ghananewsagency.org, ghanaiantimes.com.gh and graphic.com.gh) media sources. On these media platforms, we searched for articles using four keywords including Bt, GMOs, improved seeds and plant breeder.^1^ A total of 57 relevant online news articles from ghananewsagency.org (11), ghanaiantimes.com.gh (8), graphic.com.gh (10), ghanaweb.com (9), modernghana.com (5), myjoyonline.com (10) and citifmonline.com (4) were collected and analysed. Related searches conducted on the websites of AGRA media centre and Food Sovereignty Ghana (FSG) which have been active in Bt debates in Ghana to compliment the online media search yielded five articles each. Overall, article retrieval spanned from 1 January 2011, to 31 August 2016. These online news portals carried vital information concerning GMO debates in Ghana.

### Identification of GMO claims

GMO claims were identified by manually going through articles retrieved from the Ghana online media that had a bearing on debates on Bt, GMO and improved varieties. Manual content analysis involves scrutinising all material text containing claims and counterclaims relating to the topic of interest (Mkandawire, Luginaah, & Bezner-Kerr, 2011). Policy debate typically comprises a claim and supporting data, which is either explicit or implicit (Howland et al., 2006). An effective content analysis depends on the ability to identify these policy claims and supporting data. Similar to previous research, we paid attention to connecting words such as “hence”, “thus” and “because” in identifying policy claims and substance (Mkandawire et al., 2011).

### Coding of policy claims

Neuman (2005) defines coding as a set of instructions or rules on how to systematically observe and record from text. Codes are assigned to a particular text or medium of communication depending on the researcher’s interest (Mkandawire et al., 2011). For the purpose of this study, arguments were analysed in three inter-connected levels. The first level involved clustering claims that were made for and against locally produced GMOs and those imported from neighbouring countries as “local” with claims for and against the involvement of international corporations and nations excluding Ghana’s neighbouring countries as “international”. The second level separated claims in the local and international clusters into Pro-GMOs coded as “P” and anti-GMO coded as “A”. The third-level analysis assigned codes to substances (health, science, economics, environment and politics) that emerged from the content analysis. Health, science, economics, environment and politics were coded as “H”, “S”, “E”, “Et” and “Po”, respectively. The two opposing arguments were then conjoined to the five substance categories resulting in 10 coding permutations as “PH”, “PS”, “PE”, “PEt” and “PPo” for Pro-GMOs and those for Anti-GMOs include “AH”, “AS”, “AE”, “AEt” and “APo”.

Several stakeholders were involved in the GMO policy debate including local and international NGOs, members of parliament, the media, farmers associations and the Savannah Agricultural Research Institute (SARI). These diverse subsystems however, contributed unevenly to the GMO debate. As such, the study targeted active and key subsystems in the GMO policy debate. Emphasis was put on validity and rigour in data analysis. For instance, the inter-coder reliability technique was applied with two authors individually coding the same articles and comparing the results for consistency (see Mkandawire et al., 2011). Coded messages were then embedded onto original text to ensure that the derived meaning was not out of context. Having lived in Ghana for more than 20 years, the researchers have adequate knowledge of the media landscape, policy debate slants and the political economy, thus facilitating an understanding of the deeper meanings embedded in claims, counter claims and their significance for the smallholder farmer and food security.

## Results

**Figure 1.**
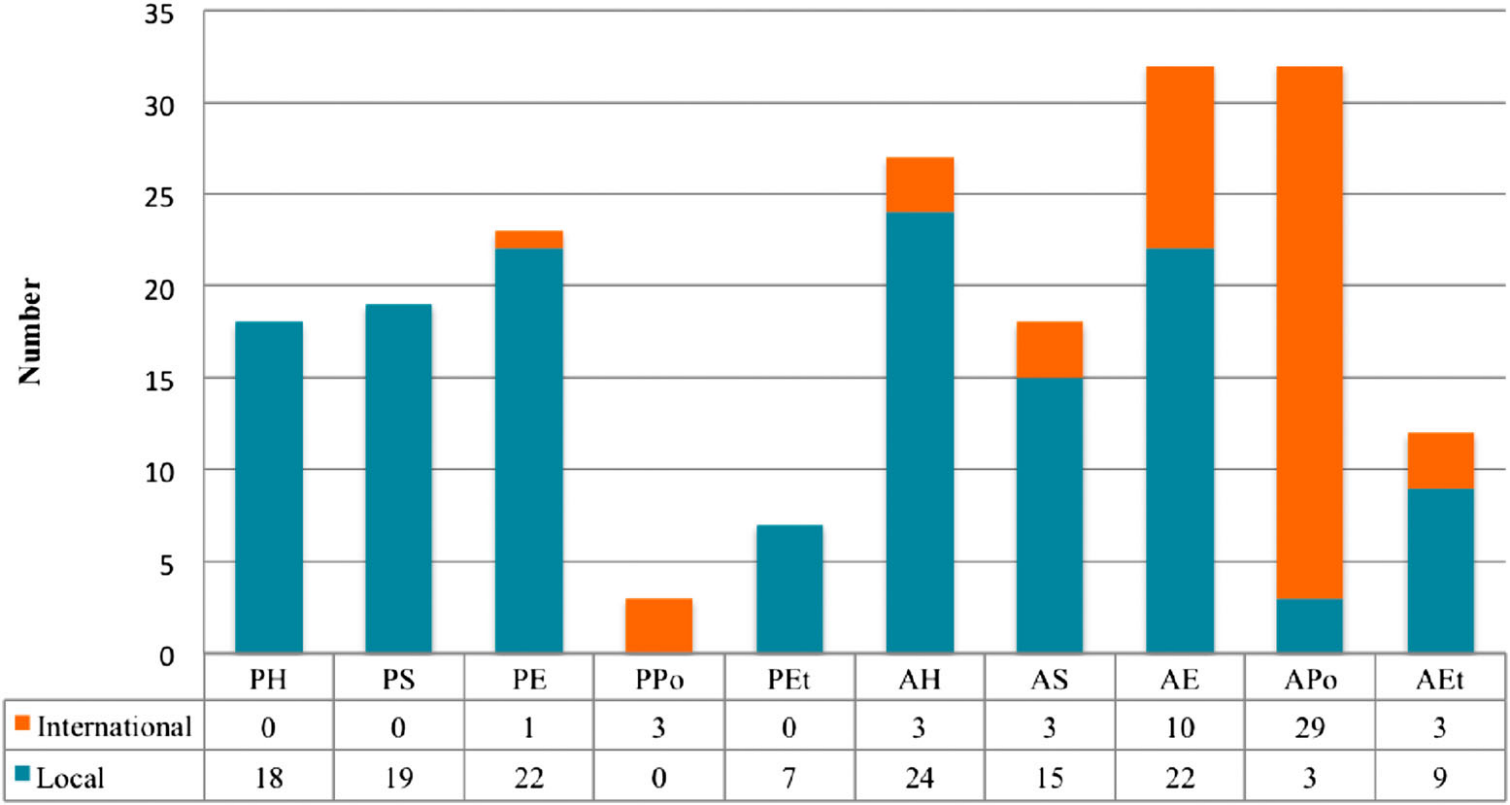
Local and international GMOs policy claims.

Following our methodological approach, pro- and anti-GMO and Bt claims were distinguished on the bases of international versus local-based claims and are shown in Table 1. Figure 1 also compares international and local policy claims separately for pro- and anti-GMO coalitions in Ghana. The Biosafety Act and the Plant Breeders Bill have been interpreted, understood and represented by the public in a number of contested ways. Anecdotal findings show little public consultation prior to the development of the Plant Breeders Bill. This has resulted in the emergence of several coalitions.

**Table 1.** GMO claims and origins in the policy debate.

Codes	Frequency	Percentage
*Reference made to GMOs origins*		
International	52	27.2
Local	139	72.8
*Coalitions*		
Pro-GMOs	70	36.6
Anti-GMOs	121	63.4
*Claims*		
Health	45	23.6
Scientific	37	19.4
Economic	55	28.8
Political	35	18.3
Environment	19	9.9

In contrast with long standing opinion that the GMO debate was about “international GMOs” and Bt, our results indicates that only about 27% of the claims referred to GMOs originating from beyond Ghana and bordering countries. Nonetheless, majority of the claims (63%) were anti-GMOs and associated with economic concerns (29%). Surprisingly, only 10% of the claims were related to the environment, suggesting a low priority on environmental concerns. In addition, we found that most of the claims from the pro-GMOs coalition were related to “local GMOs”, which probably suggests a strong support for “local GMOs” in the debate. Conversely, the anti-GMOs coalition was against both local and “international GMOs” but the majority of the claims were divided with respect to the two groups of GMOs. For instance, while health concerns were the dominant discourse in claims against “local GMOs”, politics dominated the rhetoric in claims opposing “international GMOs” (see Figure 1). These nuances indicate the complex and messy nature of the GMO debate in Ghana and possibly similar contexts. The next section presents the details of the claims presented by the pro- and anti-GMOs coalitions.

### Pro-GMOs coalition claims

The Ministry of Food and Agriculture (MoFA) envisaged the Bill to be supportive of modernisation the agriculture sector, which ultimately will improve food security. Stakeholders within the pro-GMO coalition include the Government of Ghana and its affiliated research institutions such as Council for Scientific and Industrial Research (CSIR), SARI, and some private agronomists. According to a Deputy Minister for the MoFA:“ … what the Plant Breeders Bill seeks to do is to protect breeders and not to introduce GMOs to the public” (graphic.com.gh, 10 December 2013).This is reiterated by the Chair to the Parliamentary Select Committee on Constitutional, Legal and Parliamentary Affairs when he queried that:

This has nothing to do with GMOs, [the media outlets should] stop misinforming the public and creating fear and panic  … We are talking about innovation, creativity. These are the bills that allow individuals to innovate…. Already in Ghana, plant breeding is going on. (graphic.com.gh, 15 February 2014; Member of Parliament)

As part of efforts to persuade the public to accept Bt, pro-Bt stakeholders also made science-based claims that crops produced using Bt improves yields. In this regard, a scientist within SARI argues that: “There are reports from the three northern regions that some farmers have sidestepped the regulatory system to plant Bt cotton seeds they brought in from Burkina Faso and are recording positive yields” (graphic.com.gh, 29 June 2015; Scientist, SARI).

Another pro-GMO activist, politician and trained agriculturalist stated:

The popular Nungua Black Sheep is a GMO (LMO) produced by the Legon Agriculture Research Station at Nungua in the early 1960s and remains the most widely produced in Ghana … It is highly productive … robust in terms of disease and weather resistance. (myjoyonline.com, 12 December 2013; Agriculturalist/Politician)

The Government of Ghana and its affiliate scientists hold the view that GMO products are relatively safe and environmentally friendly owing to the increased use of chemical inputs to improve yields of conventionally produced crops in recent years. Hence, they convince the Ghanaian population through the portrayal of expert forms of knowledge to accept Bt in agricultural production. A SARI scientist commented that: “Bt cowpea would not pose any danger to humans and the environment. Genetically modified (Gm) crops are sprayed at most twice unlike non-GM crops which are sprayed at least six to seven times, … ” (graphic.com.gh, 13 December 2014; Scientist, SARI). A scientist with CSIR further argued that: “Modern agricultural biotechnology … has the potential to make significant contribution to food security, poverty reduction and environmental conservation” (myjoyonline.com, 4 February, 2015; Scientist, CSIR). In spite of Ghana’s unique political and agricultural environment, pro-GMO groups use examples of GMO policies in other African countries to draw attention to the need for policy consistency and thus attempt to persuade the Ghanaian population that Bt is good and has been used successfully elsewhere. According to a Minister of Environment, Science and Technology: “In West Africa, Burkina Faso was using biotechnology for farming particularly in cotton and vegetable production, while Egypt, Kenya, South Africa and a few other African countries were already deploying genetic engineering in agriculture in various ways” (ghananewsagency.org, 30 August 2012; Minister of Environment, Science and Technology). In addition a scientist reports Bt cotton farmers in Burkina as saying: “Cultivating Bt cotton is less tedious, more yield, increased income for buying equipment and educating their children as well as taking care of their families” (ghananewsagency.org, 29 July 2015; Scientist).

According to the pro Bt stakeholders, many farmers and consumers in Ghana are already producing and consuming genetically engineered food, respectively, devoid of adverse impact on human health and the environment. Food crops from neighbouring countries like Burkina Faso produced through genetic engineering are popular in the Ghanaian market. A pro-GMO activist and scientist had this to say: “ … Ghanaian traders always go for tomatoes produced by Burkinabe farmers … although Burkina Faso practises GM agriculture … ” (myjoyonline.com, 11 February 2014; Activist and scientist). Nonetheless, these claims from the pro-GMO coalition were strongly opposed with counter claims from the anti-GMO coalition.

### The anti-GMO coalition claims

Opponents to the Bill and Bt generally focus their rhetoric on fears of adverse health effects, environmental destruction, farmer sovereignty and reduced farmer incomes. Stakeholders in this group include the Coalition for Farmers Rights and Advocacy against GMO’s (COFAM), which is an umbrella organisation formed to protect the rights of farmers. The COFAM comprises organisations such as FSG, Centre for Indigenous Knowledge for Organisational Development, Peasant Farmers Association of Ghana (PFAG), Coalition of Concerned Farmers, the Ghana Association of Farmers and Fishermen, Free the Mind Movement, Rastafarian Council, Vegetarian Association of Ghana and some opposition political parties, such as the Convention Peoples Party. Lobbying and agitations which lacked scientific backing were the main approaches employed by these coalitions. According to a Daily graphic report; “Opponents of genetically modified organisms (GMOs) and the Plant Breeders Bill have begun lobbying former members of Parliament to bring pressure to bear on current members to kick against the bill” (graphic.com.gh, 31 January 2014; Reporter). In particular, some opponents saw the Bill as an effort to introduce GMOs into the country. For instance, FSG asserts that:

there is an orchestrated attempt not only to mislead Parliament into voting for the Bill, but also to throw dust into the eyes of the Ghanaian public about the real intent and import of the Bill vis à-vis the enabling of the plant breeder to introduce GMOs into our food chain without any public awareness and participation in that decision. (ghanaweb.com, 24 January 2014; FSG)

Opponents also argued that GMOs had adverse effects on human health and their environment using scientific rhetoric. A politician with the Convention Peoples Party and anti-GMO activist made reference to a genetic scientist to have said that:

GMO’s can be toxic, allergenic or less nutritious than their natural counterparts … they do not necessarily increase yield potential; they do not reduce pesticide use but increase it; they create serious problems for farmers, including herbicide-tolerant “superweeds”; they compromise soil quality, and increase disease susceptibility in crops which have mixed economic effects; he said they harm soil quality, disrupt ecosystems, and reduce biodiversity (myjoyonline.com, 4 February 2014; Politician).

Apart from using scientific, economic and health narrative claims, the anti-GMO coalition also relies upon flaming political sentiments to rally support against GMOs. One of such activities was when the anti-GMO coalitions organised a protest march against U.S. firm Monsanto, one of the well-known multinational companies involved in Bt development. The PFAG in a press statement said;

The march against MONSANTO is to guard the sovereignty of small holder farmers in Africa over their right to save seed, sell and exchange amongst themselves  … to protect the dignity and the sovereignty of Africa;  … to protect our indigenous resources; … against greed; … for good health and vitality and  … against neo-colonialism. (modernghana.com, 24 May 2015; PFAG/Activist)

While opponents are against the passage of the Bill, which Monsanto is perceived to be the major international stakeholder and beneficiary, a similar private project known as Masara N’Arziki under the agribusiness firm Wienco, is gaining grounds in some parts of the country. Masara imports and cultivates hybrid maize and surprisingly in spite of their growing popularity, and commercials on national television, they have not captured the attention of the anti-GMO coalition. This development leaves unanswered questions regarding the real motives behind the anti-GMO movements in Ghana. Taking cue from the above assertion, we categorised the GMO policy claims into “local GMOs” focused and those relating to “international GMOs” to understand the complexities of the claims and the logic underlying the rhetoric used by different policy subsystems.

#### Local versus international GMOs

We classified claims made about Bt developed by local companies, institutions and individuals as “local GMOs”. GMO food products imported from neighbouring countries, which are often not considered international in the narratives, were also classified as “local GMOs”. SARI is one of 13 research institutes of the CSIR set up to provide small-scale farmers with appropriate technologies to increase food production. SARI has been involved in seed modification to improve crop yields. In 2011 for instance, SARI modified tomato seedlings from the International Crop Research Institute for Semi-Arid Tropics in Niger for the Ghanaian context (ghanaweb.com, 24 September 2011). It has worked closely with smallholder farmers to gain their support over the years. Their “improved seeds” as they are popularly called in the country are shown to have had a number of advantages over traditional seeds. Some farmers and consumers suggest that “improved seeds” give better yields and are more palatable. In the quote below a farmer commented that:

The new one [tomato] yields big [more], it taste nice and it last long before it goes bad. (myjoyonline.com, 25 August 2011; Farmer)

Some farmer groups and consumer coalitions in the debate were however against the business model implemented by “local GMO” companies. In their view, Bt was driven by profit, thus often pushing greater part of the risk in GMOs food production to famers. For instance, a farmer in a demonstration held a placard that read “high prices of GMO inputs are making us poorer and we farmers are the labourers and Masara N’Arziki is the beneficiary”. This reinforces farmers’ perception that all other forms but traditional plant breeding are genetically modified. Despite protests, some farmers reluctantly embraced improved seeds, pre-production financing schemes and technical support provided by “local GMO business entities” because of limited financing alternatives and generally low agricultural investment from other sources. A farmer had this to say about the owner of a seed and agro-dealer, who in collaboration with AGRA and USAID deliver improved seeds and technologies to farmers: “He helps us to buy seed and the fertilizer … we are really happy” (AGRA Media Centre). The prevalence and increased patronage of “local GMOs” has turned small-scale agriculture into a profitable business. According to a farmer: “ … market women who buy from me have even requested me to grow more of the new variety due to the favourable feedback from customers in the Tamale Metropolis” (myjoyonline.com, 25 August 2011: Farmer). Nonetheless, the assertion that GMOs tend to increase yields was still doubted by some farmers as exemplified in a statement made during a radio discussion on the potential of modified seeds:

… I decided to apply the two [varieties of] maize to see the different … To my surprise, I realized the traditional seeds performed well with good yield, far more than the GM seeds. This made [some of] the farmers in the community decide not to do anything with GM seeds. (ghanaweb.com, 26 April 2013; Farmer)

Interestingly, the debate about “international GMOs” was not about their productivity or their advantage over traditional seeds and crops. Rather, both pro- and anti-GMO coalitions employed persuasion through political narratives and appeal to emotions with limited scientific rhetoric in the debate. For instance, a pro-GMO activist and scientist in justifying the Plant Breeders Bill explained that:

… there is a lot to gain from introducing GMOs in the country because of the numerous benefits. How can we as plant breeders introduce something that will have adverse effects on our own people and the state by making it affect our exports? What is at stake is more positive than perceived. (graphic.com.gh, 14 January 2014; Scientist)

On the other hand, the anti-GMO coalition maintains that:

The ultimate result of the bill will be to put Ghana’s food supply into the hands of foreign corporations. GMOs are bullets aimed at the heart of Ghana and the plant breeders’ bill serves as the cross-hairs enabling foreign corporations to directly target Ghana, (myjoyonline.com, 17 April 2014; Coalition of Civil Society Groups against GMOs)

and

… It is strange that Ghana, Tanzania and Ethiopia have been targeted by the G8 for the accelerated proliferation of GM crops, under the guise of ensuring food security … the appointment of Kofi Annan as AGRA’s chairman was a strategic decision that the Gates Foundation made to silence criticisms that its agricultural development agenda was a White Man’s Dream for Africa. (ghanaweb.com, 11 September 2012; Columnist)

The FSG also argued that “international GMOs” would adversely affect the Ghanaian economy: “Most of the money made from Bt maize would exit Ghana, creating more wealth for foreign corporate interests, and mounting debt here in Ghana” (FSG, 21 July 2015; FSG).

Furthermore, the anti-GMO strongly suspected the pro-GMO coalition of being sponsored by large corporations and organisations to serve the business and political interests of Western countries. This position was situated within large discourses of neo-colonialism on the African continent by an anti-GMO activist and columnist:

So why are white corporations [western companies] so bent on forcing on Africa their genetically modified food seeds? Is it just for a wider market, or is there a more sinister agenda? the sad part is they are using our Ghanaian government, sponsored PhD’s, and “trusted” friend, president Obama to execute this agenda, just like they used Nkrumah’s American friend to overthrow him, resulting in the collapse of the African /black renaissance. (ghanaweb.com, 16 June 2012; Columnist)

The findings from this study suggest other complex dimensions to the debate that are different from what is already known in literature. Apart from the usual concerns about the environment, health and economic implication of GMOs, the debate in Ghana assumed a political dimension, where the anti-GMO coalitions refused to resist “local GMOs” but are vigorously fighting to stop the introduction of “international GMOs”. The main argument being that international GMOs are political tools designed to control the sovereignty of African countries (Vercillo et al., 2015). The discussion section puts these complex dimensions into the science-politics policy-making perspective within the unique context of Ghana to provide an understanding of how place-based factors and processes influence policy-making.

## Discussion

The GMO and Plant Breeders Bill debate in Ghana shows a complex interplay of science and politics claims in policy-making, influenced by socio-economic structures (Sword, 1999). This study observed that debates on Bt or GMOs stem not only from the findings from scientific studies on the environmental and health effects of genetically modified organisms, but also from individual perceptions, values and interests about the future of food and agriculture in Ghana. The different coalitions within this disparate policy subsystem resorted to narratives and rhetoric with the hope of influencing public opinion into accepting or rejecting Bt.

Scientific uncertainty about the health and environmental risks associated with plant breeding undertaken by SARI and the wholesale Bt associated with international firms like Mosanto emerged as the backbone of arguments by both sides. With each side relying on evidence, opponents claim GMOs are toxic, allergenic, less nutritious, increases pesticide use and growth of herbicide-tolerant “superweeds”, harm soil quality, disrupt ecosystems and reduce biodiversity. Proponents countered these assertions claiming that GMOs do not have adverse effects on humans and the environment. The incompleteness in science therefore helped to give rise to these contrasting claims and counter claims (Aronson, 1984). Consequently, GMO policy claims have shifted from being designative claims (based on fact) to evaluative and advocate-based claims, supported by values and actions (Garvin & Eyles, 1997). The resultant effect is a blurred distinction between politics and science in the GMO policy subsystem.

In order to back scientific claims, coalitions also resorted to economic-based claims and counter claims to persuade the public into accepting or rejecting GMOs. Capitalising on the poorly developed agricultural sector, epitomised in poor yields especially in environmentally vulnerable context such as Northern Ghana, the pro-GMO coalitions’ narratives trumpeted a boost in farm yields and farmer incomes to convince the public into accepting Bt. Conversely, anti-GMO coalitions saw the technology to be exploitative to poor farmers whose sovereignty will be taken away and manipulated by multinational corporations, thereby determining what to produce and how to produce it (Mayet, 2007). In a context where more than 70% of the population engages in peasant farming, taking away the right to determine what to produce would pose dire consequences for food security and peasantry (Ghana Statistical Service, 2013). Indeed, introducing Bt in agriculture would favour the rich and more powerful farmers to engage in export led agriculture at the detriment of poor smallholder farmers in Ghana (Mayet, 2007). Empirical findings from a recent study in the Upper West Region found that adoption of high input agriculture following the introduction of a hybrid maize technology was threatening the fragile agricultural lands, reducing yields and worsening the food security situation of smallholder farmers (Nyantakyi-Frimpong & Bezner-Kerr, 2015). On the contrary, Clottey, Karbo, and Gyasi (2009) revealed calls on various stakeholders (CSIR and MoFA) by both tomato farmers and a manufacturing company in the northern parts of Ghana to make available improved tomato varieties due to their accompanying advantages.

Additionally, economic concerns have been raised regarding the ability of farmers to export these products since Ghana’s major trading partners including the European Union have rejected GMO products. These contrasting claims and findings about the economic benefits associated with Bt lends support to individual subjective appraisal and subsequent adoption based on their perceptions, values and interests which are socially constructed (Garvin & Eyles, 1997). Consequently, the debate is much more than the science of GMOs. It touches on the identity of Ghanaians and the Ghanaian farmer who has been practising peasantry farming within a cultural step-up for decades. GMOs were therefore considered risky and threatening to existing socially and culturally constructed conventional forms of farming. This could probably explain why “local GMOs” are not resisted as much as “international GMOs” in the debate which has important relevance in improving agriculture in Ghana and other developing countries.

Invariably, we observe the extensive use of food security as the scientific rhetoric in both pro- and anti-GMO coalitions. Food security has been one of the development concerns of the country and therefore each coalition found it worthwhile to frame the arguments in the context of food security. Despite significant improvement at the national level, culminating in Ghana being declared as one of the few countries in SSA to have achieved the United Nations Millennium Development Goal of halving the proportion of people suffering from hunger (FAO et al., 2015), there are marked in-country disparities. For instance, while the Global Hunger Index, a multi-dimensional hunger measure was 89% in 2012 at the national level (WFP & MoFA, 2012), a recent study in the Upper West Region in 2014 suggests that 63% of households are severely food insecure (Atuoye, 2015). Some of these examples of worsening food insecurity became the focal point for claims making and rebuttals leading to an intense debate on the best strategy to address such extreme food insecurity. While pro-GMO favour Bt food production and high input agriculture, the anti-GMO supported ecological agriculture, reflecting the long running debate about agricultural intensification and agro-ecological farming (Bezner-Kerr, Nyantakyi-Frimpong, Lupafya, Dakishoni, & Organization, S. 2016).

The debate showed low farmer participation and consultation in the policy-making process contributing to a skewed elite’s position in the proposed policy, where the focus was on the benefit of the Bill to seed growers and breeders and little consideration about the benefit and risks to the peasant farmer. This reflects a general non-consultation of grassroots population in policy-making in many developing countries. For a successful and informed policy-making, education and awareness creation are cardinal in offering individuals the opportunity to evaluate and understand innovations for informed decision-making (Deffor, 2014). Rowe (2004) established a positive correlation between education and GMO resistance. Furthermore, a study by Zakaria (2014) indicates an association between farmers’ basic knowledge of GMOs and its adoption in a study of 305 members of farmer-based organisations in the Northern Region of Ghana. Accordingly, educating farmers on Bt and granting them equal opportunity and voice in the policy debate will bring to bear farmer experiences on the positives and negatives of Bt. Moreover, a blend of farmer experiences with scientific evidences will help clear much of the uncertainties surrounding the technology thereby improving the policy-making space for effective engagement between the subsystems.

## Conclusion

Similar to normal practice in policy-making, the Plant Breeders Bill in Ghana is going through intense and highly contested debate with subsystems forming pro- and anti-GMO coalitions to engage each other with rhetorical claims. Uncertainty over the health and environmental impacts of GMOs and their potential for increasing yields in the context of Ghana has promoted long drawn claims making using science and politics. Not only have these claims left many in a state of confusion, it has also stalled the passage of the Plant Breeders Bill in Ghana. Indeed, the GMO policy debate can be described as a messy business (Pal, 1992) considering the complex interplay of contrasting scientific findings and values used to pursue policy claims. Whereas food security was used as the basis for claims making, both coalitions were also influenced by interest and values emanating from strong association to profit making and neoliberalism on one hand, and sovereignty and protectionism on the other hand.

The study found three unique features with the policy debate in Ghana that are relevant for food security and agricultural policy-making in the country and elsewhere. First, claims making in the policy debate was without critical perspectives from smallholder farmers who are the bulk of farmers in the country. Without this, the debate remained an elitist forum, which was probably devoid of complex social and cultural dimensions of local agricultural systems and their inter-connectedness with food culture and food security. Second, resistance of GMOs is more complex and selective between international and “local GMOs”. The feeling of “This Is Mine” (TIM) and “That Is Theirs” (TIT) were stronger in mobilising support for “local GMOs” and resistance for “international GMOs” than scientific and economic considerations. In considering policies for agricultural innovation and food security strategies, TIM and TIT highlight a strong need for the role of context. Third, absorption of GMOs in developing countries invariably requires high input agriculture which likely cut out poor farmers. Worse of all, large scale agriculture takes away lands and space from many smallholder farmers which has dire consequences for food security among local populations (Li, 2011). We recommend that food security and agricultural policy-making in Ghana and other SSA countries need to pay attention to the three points raised here to avoid the collapse of peasantry.

## Note

1. We observed from the debates in Ghana that GMOs, biotechnology, plant breeders and improved varieties are referred to as the same. Hence, in this paper, we treat the terms as the same.
